# Genome-wide identification of Aux/IAA gene family and their expression analysis in *Prunus mume*

**DOI:** 10.3389/fgene.2022.1013822

**Published:** 2022-10-12

**Authors:** Wenhui Cheng, Man Zhang, Tangren Cheng, Jia Wang, Qixiang Zhang

**Affiliations:** Key Laboratory of Genetics and Breeding in Forest Trees and Ornamental Plants of Ministry of Education, School of Landscape Architecture, Beijing Forestry University, Beijing, China

**Keywords:** Aux/IAA gene family, *Prunus mume*, evolutionary analysis, expression pattern analysis, auxin-responsive genes

## Abstract

*AUXIN/INDOLE ACETIC ACIDs* (Aux/IAAs), an early auxin-responsive gene family, is important for plant growth and development. To fully comprehend the character of *Aux/IAA* genes in woody plants, we identified 19 *PmIAA* genes in *Prunus mume* and dissected their protein domains, phylogenetic relationship, gene structure, promoter, and expression patterns during floral bud flushing, auxin response, and abiotic stress response. The study showed that PmIAA proteins shared conserved Aux/IAA domain, but differed in protein motif composition. 19 *PmIAA* genes were divided into six groups (Groups Ⅰ to Ⅵ) based on phylogenetic analysis. The gene duplication analysis showed that segmental and dispersed duplication greatly influenced the expansion of *PmIAA* genes. Moreover, we identified and classified the cis-elements of *PmIAA* gene promoters and detected elements that are related to phytohormone responses and abiotic stress responses. With expression pattern analysis, we observed the auxin-responsive expression of *PmIAA5, PmIAA17,* and *PmIAA18* in flower bud, stem, and leaf tissues. *PmIAA5, PmIAA13, PmIAA14, and PmIAA18* were possibly involved in abiotic stress responses in *P. mume*. In general, these results laid the theoretical foundation for elaborating the functions of *Aux/IAA* genes in perennial woody plant development.

## Introduction

Auxin, as one of the essential hormones for plant growth, is engaged in the longitudinal growth of vegetative organs by promoting cell elongation and growth (Weijers and Wagner, 2016). Early auxin response genes usually refer to genes whose expression was induced reaching maximum level within several minutes to a few hours after auxin treatment (Hagen and Guilfoyle, 2002). In-plant species, the early auxin-responsive genes are primarily consisted of three gene families: *Aux/IAAs*, *GRETCHEN HAGEN3s* (GH3s), and *SMALL AUXIN UP RNAs* (SAURs) (Ren and Gray, 2015). Aux/IAA proteins is a set of plant-specific short-lived proteins located in the cell nucleus (Abel et al., 1994). The *Aux/IAA* genes encode proteins of molecular weights ranging from 20 to 35 kDa, which are usually degraded via the 26S proteasome pathway (Smalle and Vierstra, 2004). *Aux/IAA* genes were firstly reported to regulate hypocotyl elongation in *Glycine* max (Walker and Key, 1982). It is shown that Aux/IAA proteins usually have four protein conserved domains (domain I to Ⅳ) with two protein peptides used as nuclear localization signals (Dargeviciute et al., 1998). Among four domains, domain I contained a conserved ERF-associated motif, which recruited co-suppressor TOPLESS (TPL) proteins to regulate the transcriptional activity of the Auxin Response Factor (ARFs) (Tiwari et al., 2004). Domain Ⅱ, essential in auxin signaling transduction, contained 13 specific amino acid core sequences ‘VGWPP’ that constitute a degradation unit targeted by the ubiquitin-ligase SCF^TIR1^ (Ouellet et al., 2001; Dharmasiri et al., 2003). Domain III can dimerize *in vitro* and have a turning-helix-helix (βαα) region (Morgan et al., 1999). Domain Ⅲ and Domain Ⅳ are homologous to the C-terminal domain of ARFs, which enables Aux/IAA to dimerize or polymerize and bind ARFs to inhibit their functions (Woodward and Bartel, 2005).

Many studies have reported the functional roles of *Aux/IAA* genes in model plants, including regulating root, stem, leaf, and fruit development (Guilfoyle, 2015). An auxin-resistant Arabidopsis mutant *atiaa28* has shown serious defects in lateral root formation, indicating that *AtIAA28* represses the transcription of initial genes in lateral roots (Rogg et al., 2001). Moreover, *OsIAA11, OsIAA13,* and *OsIAA30* regulate the expression of lateral root formation genes and repress lateral root growth in rice by auxin signal transduction (Kitomi et al., 2012; Zhu et al., 2012). In potatoes, the down-regulation of *StIAA2* led to increased plant height, hyponastic petiole, and extremely bendable growing leaf primordia at the shoot apex, indicating that *StIAA2* was involved in the development of stems and leaves (Kloosterman et al., 2006). On the other hand, *StIAA9* was highly expressed in the germination and expansion of potato tubers (Gao et al., 2016). *Aux/IAA* genes also participated in regulating plant reproductive organ development, such as fruit formation and flower development. Qiao et al. found that OsARF6 interacts with OsIAA8 and OsIAA20 to negatively regulate rice grain length (Qiao et al., 2021). The silencing of *SlIAA17* in tomatoes can result in larger fruit size and increased skin thickness by affecting cell size (Su et al., 2014; Su et al., 2015). Additionally, *Aux/IAA* genes also played vital roles in abiotic stress response. For instance, the loss of *AtIAA5/6/19* led to decreased glucosinolates (GLSs) levels in plants exposed to drought, thereby causing reduced drought tolerance (Salehin et al., 2019). Under high salinity conditions, *OsIAA9* and *OsIAA20* were significantly up-regulated (Jain and Khurana, 2009). Furthermore, *OsIAA20* overexpression transgenic rice showed increased drought and salt tolerance, while the *OsIAA20* RNAi transgenic plants showed opposite phenotype, suggesting *OsIAA20* can improve plant adaption to abiotic stress in rice (Zhang A. Y. et al., 2021).

Three types of regulatory factors, including ARF, Aux/IAA, and Transport Inhibitor Response one/Auxin Signaling F-box (TIR1/AFB), were related to auxin signal transduction (Mockaitis and Estelle, 2008; Weijers and Wagner, 2016). Aux/IAA proteins mainly act through two functional pathways. The classic Aux/IAA proteins can form Aux/IAA-AUXIN-TIR1/AFB co-receptors through recognition and binding of TIR1/AFB (Guilfoyle and Hagen, 2007). SCF is a ubiquitin-protein ligase complex consisting of three subunits (Skp1, Cullin, and F-box proteins) (Smalle and Vierstra, 2004). As auxin increased to high levels, Aux/IAA-AUXIN-TIR1/AFB protein complex enters SCF through interaction with the C-terminal domain of F-Box protein, forming SCF^TIR1^-AUXIN -Aux/IAA complex (Smalle and Vierstra, 2004). The degradation of Aux/IAA by ubiquitination brought about the release of ARF proteins from the complex to make it bind the cis-acting elements of downstream genes (Smalle and Vierstra, 2004). On the other hand, the non-classical Aux/IAA proteins, such as AtIAA32 to AtIAA34, cannot be recognized by TIR1/AFB receptors due to the missing domain II. These Aux/IAA proteins function via the TMK1-IAA32/34-ARF pathway, which is a novel auxin signaling pathway proposed recently (Cao et al., 2019). Moreover, Lv et al. found that IAA33, acting downstream of MPK14 (Mitogen-Activated PROTEIN kinase 14), can function through the MPK14-IAA33-ARFs pathway that is parallel to Aux/IAA-TIR1-ARFS signal transduction pathway (Lv et al., 2020).

Mei (*Prunus mume*) is a significant perennial woody ornamental flower classified in the *Prunus* genus of the Rosaceae family and is famous for its early blooming and unique fragrance (Zhuo et al., 2021). Earlier reports have explored the auxin-responsive *ARF* family genes in mei (Song et al., 2015). Despite this, the roles of the *Aux/IAA* genes in the development of mei have not been elucidated. Our study conducted a genome-wide survey for *Aux/IAA* gene family in mei and analyzed their gene structure, protein property, and phylogenetic relationships. Subsequently, we performed genome synteny analysis to infer the duplication mode and evolutionary trajectory of *PmIAA* genes in mei. Furthermore, we analyzed the expression patterns of *PmIAAs* across distinct organs and during the process of flower bud development, hormonal regulation, and stress response to understand their functional roles. This research offered a theoretic basis for the functional examination of *PmIAA* genes in regulating floral bud development, hormonal response, and abiotic stress response in mei, which will further inform relevant studies about *Aux/IAA* genes in other perennial woody plants.

## Materials and methods

### Genome-wide classification for *PmIAA* gene family in mei

The whole-genome sequences of *Prunus persica*, *Populus trifoliata*, *Arabidopsis thaliana,* and *Oryza sativa* were downloaded from the Phytozome v13.0 online website, respectively. The genome of mei was obtained from the mei genome database (Zhang et al., 2012). To identify *Aux/IAA* family genes, we obtained the hidden Markov model file (Pfam ID: PF02309) from the Pfam database based on the conserved protein domain of *Aux/IAA* gene family proteins (Mistry et al., 2021). We searched for genes containing Aux/IAA featured domains among genomes of five plant species using the HMMER Search tool implemented in HMMER3.0 software and considered candidate *Aux/IAA* family members with an e-value < 1e^−10^. To validate the integrity of conserved protein domains, we used the Conserved Domains Search Tool from NCBI and the SMART online tool (Letunic et al., 2021) to analyze the conserved protein domains of *Aux/IAA* family members from five species. Moreover, we aligned *PmIAA* proteins using the ClustaIW algorithm implemented in MEGA11 software (Tamura et al., 2021). The protein sequence alignment was visualized with GeneDoc software (Nicholas, 1997).

### Gene structure and protein motif analysis of *PmIAA* genes

The gene structure information regarding to the exon and intron coordinates was obtained and analyzed for *PmIAA* genes (Chen et al., 2020). We then mapped *PmIAA* genes across chromosomes using MapChart2.0 software and renamed *PmIAA* genes according to their chromosomal location. Furthermore, we identified protein motifs of *PmIAA* proteins using the MEME online tool by only considering motifs with an e-value <0.05 and using the PROSITE database (Bailey and Elkan, 1994). The protein motifs and gene structures of *PmIAAs* were visualized by the TBtools software. Finally, we analyzed the biochemical property of PmIAA proteins using ProtParam Expasy online software tools and obtained their molecular weight, isoelectric point, and hydrophilic properties (Duvaud et al., 2021). The plant-Ploc Server online tool was used to forecast the cellular localization of PmIAA proteins (Chou and Shen, 2008).

### Phylogenetic tree construction of *Aux/IAA* genes

To understand the phylogenetic correlation of Aux/IAA proteins among five plants, we first aligned the protein sequences of *Aux/IAA* gene family members using the ClustaIW algorithm implemented in MEGA11 with default parameters (gap opening penalty = 10; gap extension penalty = 0.2 in multiple alignments; delay divergent cutoff = 30%). We constructed a phylogenetic tree based on the ML (Maximum Likelihood) method with a bootstrap of 1000 replicates and visualized using the iTOL tool (https://itol.embl.de/) (Letunic and Bork, 2021).

### Microsynteny analysis of *PmIAA* genes

To understand the gene duplication types of *PmIAAs*, we self-blasted the genome of mei and assigned genes into different categories utilizing MCScanX (Wang et al., 2012). To further infer the syntenic relationship among *Aux/IAA* family genes of five plants, we performed all-to-all blast on Aux/IAA protein sequences of five species followed by interspecies colinear analysis using MCScanX. The collinear blocks were extracted and the syntenic relationship of *Aux/IAA* genes was visualized using the “Multiple Synteny Plot” function of TBtools software.

### Cis-acting element inquiry of *PmIAA* promoter sequences

We extracted 2kb sequences upstream of the start codon of *PmIAA* genes as their promoter sequences. The cis-acting elements of *PmIAA* promoters were identified via the PlantCARE tool (Lescot et al., 2002). The key elements were further classified and summarized by ‘tidyverse’ packages in R.

### Expression pattern analysis of *PmIAA* genes

To distinguish the expression pattern of *PmIAAs* across different organs and during floral bud dormancy process, we first obtained the RNA-seq data from the NCBI SRA database (accession number: GSE40162 and PRJCA000291, respectively), which was preprocessed and normalized to obtain FPKM values of *PmIAA* genes following standard transcriptome analysis pipelines (Zhang et al., 2021a). The expression pattern of *PmIAAs* across tissues was further visualized using the ‘heatmap’ function in R.

### Plant material and treatments

The mei tree used in this study is cultivated in Beijing Forestry University (China). To examine the expression of *PmIAAs* in hormonal and abiotic stress treatments, we first applied exogenous plant hormones (100 μmol/L IAA) on floral buds after dormancy release and on newly sprouted branches in spring. The control group was sprayed with water. The floral buds, stems, and leaves (within the first three internodes) from the treatment and control group were gathered at 0, 1, 3, 6, 12, and 24 h after the IAA treatments. Similarly, the salt stress treatment (160 mmol/L NaCl) and drought stress treatment (20% PEG) was applied to the newly sprouted branches with the control group treated with water. Immature leaves and stems were collected at 0, 1, 3, 6, 12, and 24 h after abiotic stress treatment. All gathered samples of each treatment with a set of three biological replicates were frozen in liquid nitrogen and stored in -80°C.

### Total RNA extraction and quantitative real-time PCR (qRT-PCR)

To analyze the relative expression levels of *PmIAAs* across samples*,* we first extracted the total RNA from leaves, stems, and buds with the RNA extraction kit (OMEGA, China) and examined the concentration and quality of RNA. Then we synthesize first-strand cDNA with 1 μg total RNA by the means of the PrimeScript RT Reagent kit (TaKaRa, China). We made use of synthesized cDNA to carry out qRT-PCR by using the SYBR Premix Ex Taq II kit (TaKaRa, China). Furthermore, qRT-PCR analysis needs a reference gene (*PmPP2A*) based on earlier studies, and primers of the qRT-PCR program were designed by the NCBI Primer designing online tool (Supplementary Table S1) (Zhang et al., 2021b). The qRT-PCR setting is in this way: 95°C for 30 s; 35 cycles of 95°C for 5 s; 60°C for 15 s; 72°C for 5 s. Three biological replicates were set for each gene. We then calculated the gene relative expression quantity through the 2^−ΔΔCt^ method (Schmittgen and Livak, 2008). Finally, the relative gene expression levels obtained were visualized based on the ‘ggplot2’ function in R. Multiple comparisons of relative gene expression levels were performed using Fisher’s Least Significant Difference (LSD) method by ‘Agricola’ package in R.

## Results

### Genome identification of *Aux/IAA* genes in *P. mume*

The Aux/IAA conserved profile was used to obtain all *Aux/IAA* genes in the mei using an HMMER Search tool. We detected 31 putative *Aux/IAA* genes in total containing Aux/IAA conserved domains with e-value≤1e^−10^ (Supplementary Table S2). We further confirmed the integrity of the Aux/IAA protein domain using the Conserved Domain database tools and SMART online tools and excluded those with no complete Aux/IAA domains. Finally, we obtained 19 correct *Aux/IAA* family genes and renamed them from *PmIAA1* to *PmIAA19* according to their chromosome location. All 19 *PmIAA* genes are distributed on five chromosomes including chromosome 1, chromosome 2, chromosome 4, chromosome 6, and chromosome 8 (Supplementary Figure S1). Among them, chromosome two and four contain six and five *PmIAA* genes, respectively. Other chromosomes each contains two to three *PmIAA* members. 19 *PmIAA* genes encode proteins of 190 (*PmIAA13*) to 489 amino acids (*PmIAA11*) (Supplementary Table S1). By analyzing protein biochemical properties, we observed that the molecular weights of PmIAA proteins range from 12.88 to 54.99kDa (Supplementary Table S2). The theoretically charged coefficients of PmIAA proteins ranged from 5.52 (PmIAA7) to 9.34pI (PmIAA11) and the gravy ranked from -0.771 (PmIAA16) to -0.255 (PmIAA6) (Supplementary Table S3). Moreover, the cellular localization of all PmIAA proteins was predicted as the nucleus (Supplementary Table S4).

### Multiple sequence alignment and conserved domains analysis of *PmIAA* genes

**FIGURE 1:**
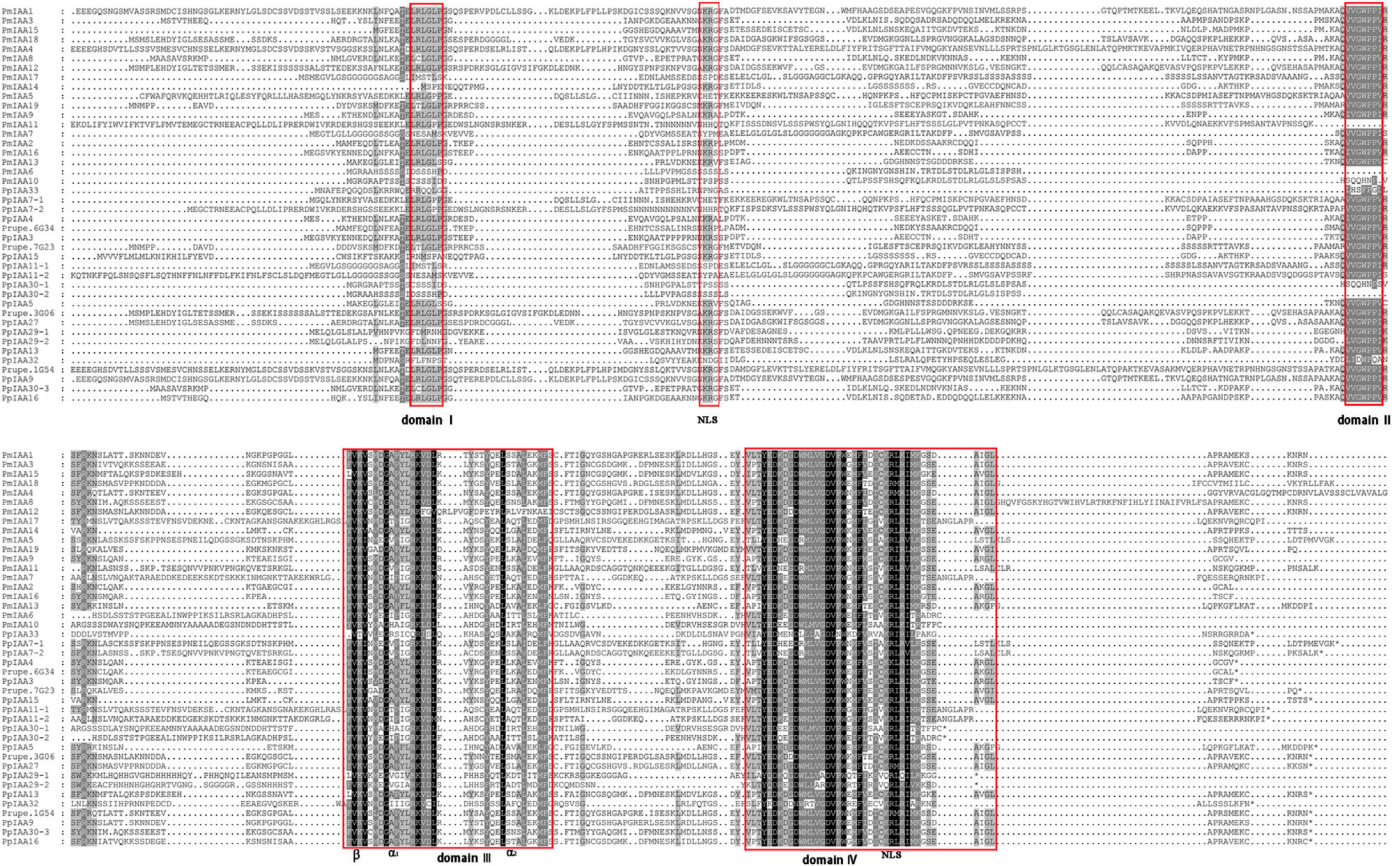
Sequence alignment and conserved domains analysis between PmIAA proteins and PpIAA proteins. Domain I to domain IV of the PmIAA proteins are indicated with black words and red lines. Similar and conserved amino acid residues were represented by the color shading.

In the pairwise alignment of PmIAA protein, we observed that amino acid sequence similarity between any two PmIAAs was typically between 30% and 60%, with the least identity between PmIAA1 and PmIAA10 (28.77%) and the highest between PmIAA9 and PmIAA16 (64.92%) (Supplementary Table S5). 19 PmIAA proteins were aligned via the ClustalW program with 23 PpIAA proteins used as reference. The alignment showed that most PmIAA proteins have four conserved domains (domain Ⅰ to Ⅳ) (Figure 1). 14 PmIAA proteins contain the classic ‘LxLxLx’ motifs within domain I except for PmIAA6, PmIAA7, PmIAA10, PmIAA14, and PmIAA17 (Figure 1). The protein motif ‘VGWPP’ within domain Ⅱ is related to protein degradation and is crucial to protein stability of proteins (Ouellet et al., 2001). However, the ‘VGWPP’ motif was not detected within PmIAA6, PmIAA10, and PmIAA11. All Aux/IAA family proteins share the complete protein domains Ⅲ and Ⅳ. Domain Ⅲ usually consists of three parts (β, α1, and α2). However, the α1 and α2 conserved sequences of PmIAA12 were less consistent than that of the other Aux/IAA proteins. Since Aux/IAA are short-lived in the nucleus, most PmIAA proteins possessed two NLSs. The first NLS is a dichotic ‘KR’ motif located between domain Ⅰ and domain Ⅱ, while the second NLS is a conserved ‘KRLR’ motif located in Domain Ⅳ (Guan et al., 2019). 13 PmIAA proteins contain two NLS signals except that PmIAA5, PmIAA6, PmIAA7, PmIAA10, PmIAA11, and PmIAA17 have only one NLS signal next to domain Ⅳ (Figure 1).

### Construction of phylogenetic tree in five plant species

**FIGURE 2:**
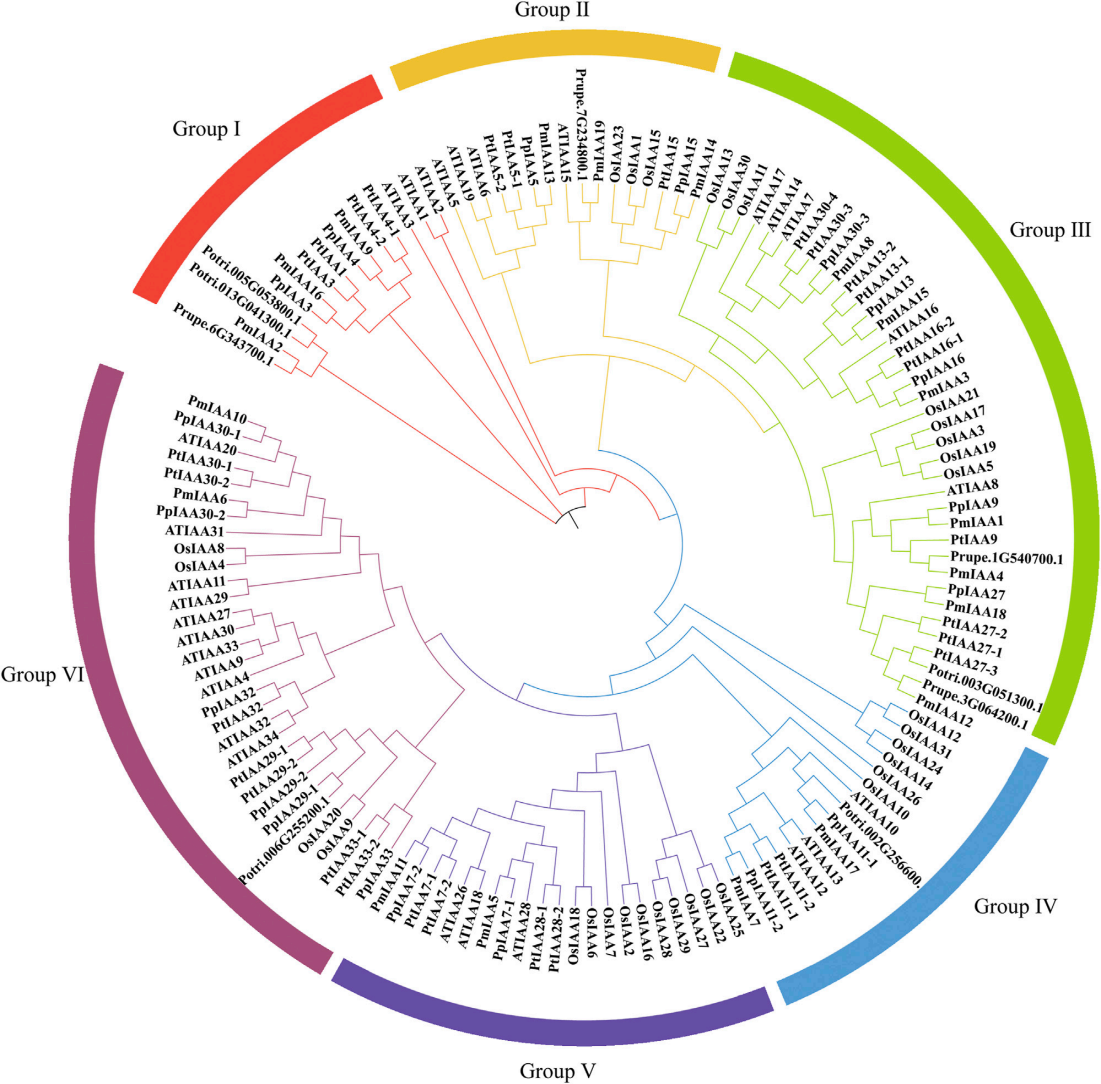
Construction of Phylogenetic tree for Aux/IAA proteins among mei, peach, poplar, Arabidopsis, and rice. Protein sequences were aligned by the ClustalW and the phylogenetic tree was constructed with MEGA 11 software through the ML method. The six Aux/IAA groups (Ⅰ–Ⅵ) were colored differently.

In total, we detected 23, 35, 29, and 31 Aux/IAA members in peach, poplar, Arabidopsis, and rice, respectively. For dissecting the phylogenetic relationship of Aux/IAA proteins, we constructed phylogenetic trees of all 137 Aux/IAA proteins (Supplementary Table S4). Among all five species, Aux/IAA proteins of mei and peach were the most closely related, followed by poplar, Arabidopsis, and rice. All Aux/IAA proteins were divided into six groups (Group I to Group Ⅵ) (Figure 2). However, we found that Aux/IAA proteins from Group I were absent from rice (Figure 2). Mei, peach, and Arabidopsis all contained three Aux/IAA proteins, but poplar had six proteins (Figure 2). As for Group II, the other four species except Arabidopsis contain three Aux/IAA proteins (Figure 2). In Group III, the number of proteins in Arabidopsis, poplar, and rice are five, eleven, and eight, respectively, while there are seven in peach and mei (Figure 2). For Group IV, Arabidopsis, mei, poplar, and peach all contained two to three proteins but rice contained five proteins (Figure 2). In Group V, the number of Aux/IAA proteins in rice is up to eight, and the number of proteins in other species is between two to four (Figure 2). As for Group VI, the number of proteins varies greatly among species. Arabidopsis and poplar have eleven and eight Aux/IAA proteins within Group VI, respectively. However, there are five proteins in peach, five in Arabidopsis, and only two in mei (Figure 2). The amount of protein in peach is the same as mei except that in Group VI. Among woody plants, compared with peach and mei, we found that the Aux/IAA protein of each group in poplar was several more than that of peach and mei, especially for Group I, Group III, and Group VI.

### Gene structure and protein motif analysis of *PmIAAs*

**FIGURE 3:**
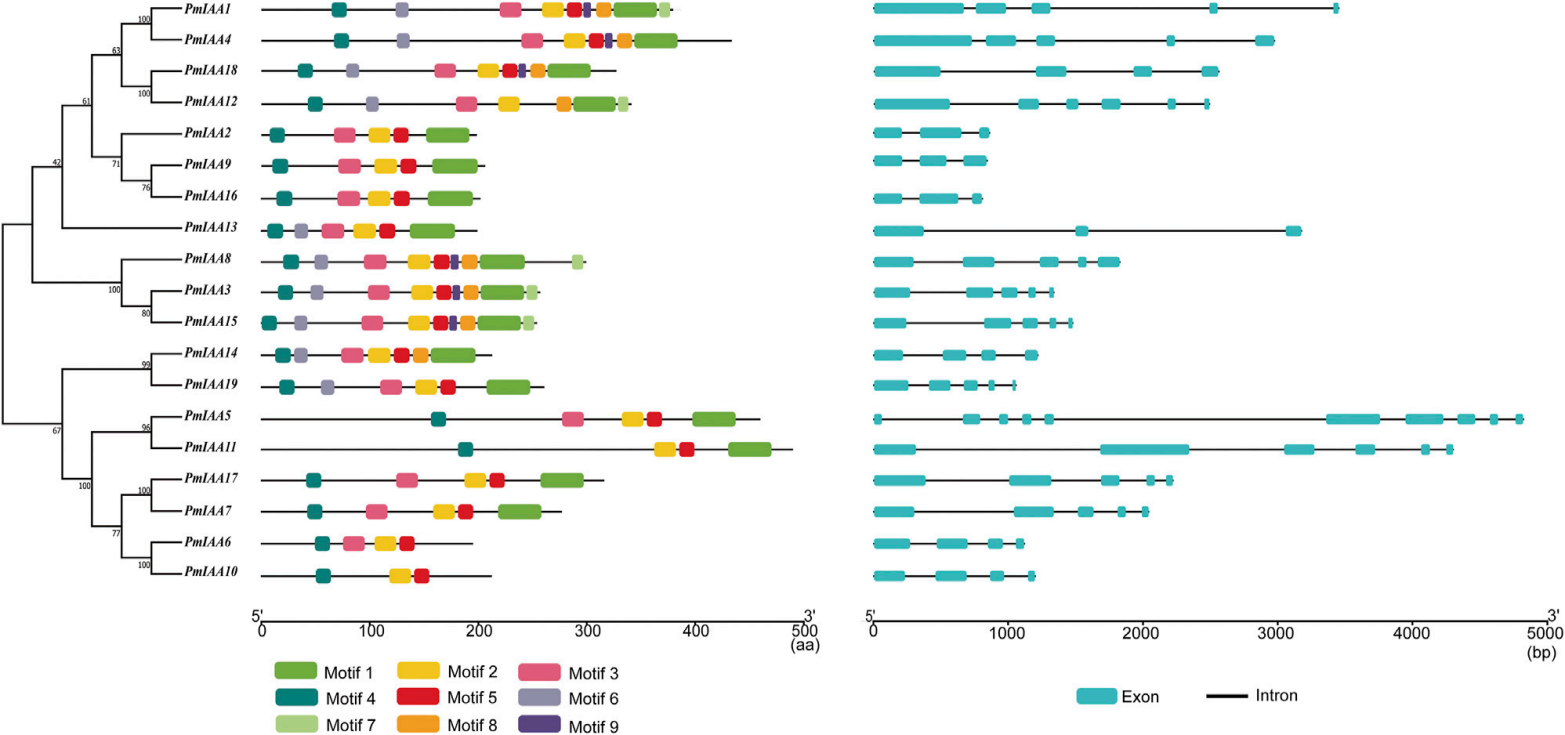
The phylogenetic relationship, protein motif, and gene structure analysis of *PmIAAs* in mei. The ML tree on the left includes 19 PmIAA proteins. The schematic in the middle represents different protein motifs identified using MEME. The gene structure of PmIAAs was displayed on the right.

We analyzed the gene structural composition of Aux/IAA family members in mei and observed that all *PmIAA* genes contained at least three exons (Supplementary Table S2). Among 19 *PmIAA* genes, *PmIAA5* contained the greatest number of exons (10 exons) (Figure 3). 12 *PmIAA* genes are composed of three or four introns with conserved positions (Figure 3). We also detected a reduced number of introns within some *PmIAAs,* including *PmIAA2*, *PmIAA9, PmIAA13*, and *PmIAA16,* and an increased number of introns within *PmIAA5* and *PmIAA11* (Figure 3). In general, the exon and intron distribution of *PmIAA* genes were conserved among plant species. Additionally, nine conserved motifs were identified in PmIAA family proteins with an e-value ≤ 0.05. More than 17 PmIAA proteins contained motif 1, motif 2, motif 3, motif 4, and motif 5 (Figure 3, Supplementary Table S6 and Supplementary Figure S2). Motif one to motif four were distributed within the conserved Aux/IAA domains (Figure 3). As for other protein motifs, there are five, six, eight, and ten PmIAA proteins contain motif 7, motif 9, motif 8, and motif 6, respectively. Furthermore, four PmIAA proteins (PmIAA1, PmIAA3, PmIAA8, and PmIAA15) have all nine protein motifs.

### Analysis of cis-elements in *PmIAA* gene promoters

**FIGURE 4:**
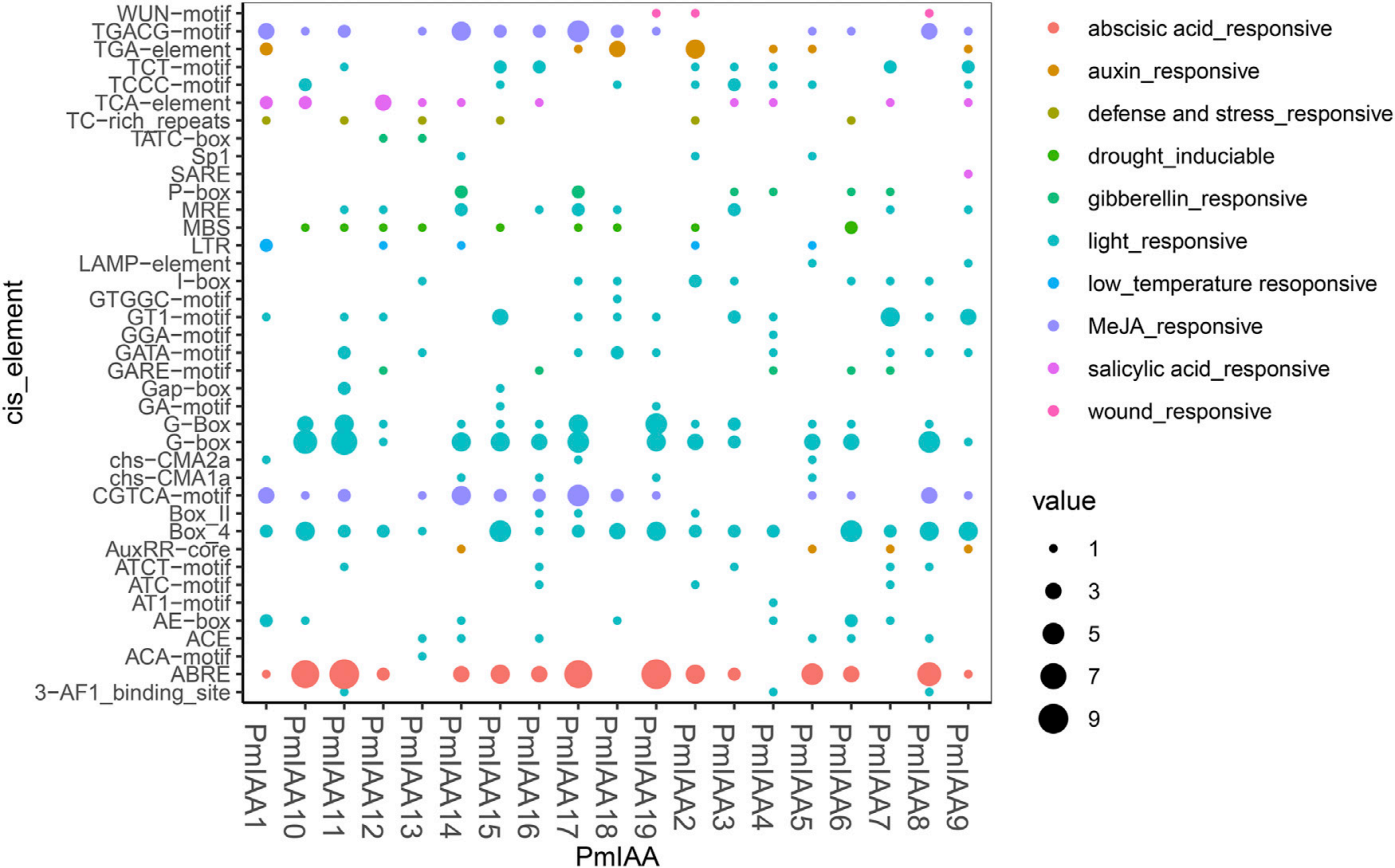
Promoter sequence analysis reveals the functional cis-acting elements involved in stress-responsive and hormonal signaling. The *x*-axis represents *PmIAA* genes and the *y*-axis indicates the number of each kind of cis-acting element within each *PmIAA* gene.

To study the regulatory mechanism of *PmIAA* genes, we extracted the 2kb promoter sequences upstream of the translation beginning site of *PmIAAs* and predicted the cis-acting elements using the PlantCARE tool. We mainly focused on the predicted cis-acting elements related to environmental stresses and hormonal responses (Supplementary Tables S8 and S9). We observed that all 19 *PmIAAs* shared light-responsive elements, including ATCT-motif, GATA-motif, AE-box, Box-4. *PmIAA11* promoter region contained the greatest number of light-responsive elements up to 22, while *PmIAA1* only has four (Figure 4; Supplementary Table S10). For stress-related elements, low-temperature responsive cis-acting element (LTR element), defense and stress response cis-acting element (TC-rich repeats motif), drought-inducible MYB binding site (MBS element), and wound response element (WUN-motif) were observed in the promoters of five, six, nine, and three *PmIAA* genes, respectively (Figure 4; Supplementary Table S11). A total of 172 cis-acting elements associated with hormonal response were found among *PmIAA* gene promoters (Figure 4; Supplementary Tables S12 and S13). Among 19 *PmIAAs*, 15 *PmIAAs* had abscisic acid response cis-acting element (ABRE element), 14 *PmIAAs* contained methyl jasmonate responsiveness cis-acting regulatory element (CGTCA-motif and TGACG-motif), and 10 *PmIAAs* contained salicylic acid response cis-acting elements (TCA-element and SARE element) (Figure 4; Supplementary Table S14 and S15). Additionally, auxin response element (TGA-element and AuxRR-core motif) and gibberellin response element (P-box, GARE-motif, and TATC-box) were present in nine and eight *PmIAA* gene promoters, respectively (Figure 4; Supplementary Table S16 and S17). We also found that *PmIAA10, PmIAA11, PmIAA17,* and *PmIAA19* contained eight or nine ABRE elements, which is the largest number of single elements, and *PmIAA10* and *PmIAA11* contained up to six to seven G-box elements, which are light-responsive (Figure 4, Supplementary Table S18).

### Expression pattern analysis of *PmIAAs* across diverse organs and bud development process

**FIGURE 5:**
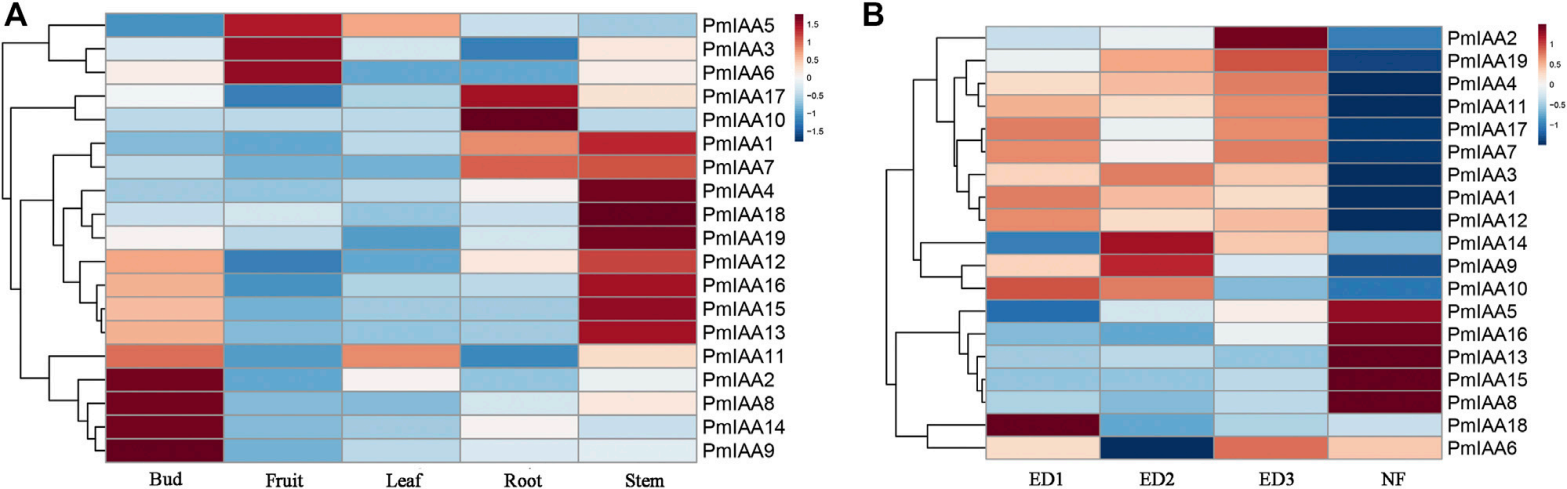
Expression pattern analysis of *PmIAAs* during five organs and floral bud development **(A)** The gene expression pattern of *PmIAA* genes in root, stem, leaf, floral bud, and fruit tissues **(B)** Expression pattern of *PmIAA* genes during floral bud flush. Expression trends of *PmIAA* in the dormancy release of floral bud. ED one and ED two represent different degrees of endodormancy, ED three represents eco-dormancy, and NF represents flower bud break.

We observed differentiated expression patterns of *PmIAAs* across five organs (leaf, flower bud, fruit, root, and stem), indicating their functions at a certain stage of plant organ development. Among all *PmIAAs*, we observed that the expression levels of nine genes (*PmIAA2, PmIAA8, PmIAA9, PmIAA11, PmIAA12, PmIAA13, PmIAA14, PmIAA15,* and *PmIAA16*) were relatively high in flower buds (Figure 5A). *PmIAA3, PmIAA5* and *PmIAA6* were relatively high-expressed in fruits (Figure 5A). However, only *PmIAA11* and *PmIAA5* were relatively high-expressed in leaves. In addition, *PmIAA1, PmIAA7, PmIAA10,* and *PmIAA17* were relatively high-expressed in roots and the expression level of *PmIAA1*, *PmIAA4, PmIAA7, PmIAA12, PmIAA13, PmIAA15, PmIAA16, PmIAA18,* and *PmIAA19* were relatively high in stems (Figure 5A). 

The trends of *PmIAA* gene expression levels during the flower bud dormancy release process were also analyzed to further explore the functional roles of *PmIAAs* in regulating floral bud dormancy release (Zhang et al., 2018). We observed that the *PmIAA* genes can be generally clustered into five groups based on their expression levels trend. *PmIAA5, PmIAA8, PmIAA13, PmIAA15,* and *PmIAA16* were relatively low-expressed from endodormancy (ED1) to eco-dormancy (ED3) and were induced significantly during bud flush (Figure 5B). However, the expression levels of *PmIAA1, PmIAA10, PmIAA12,* and *PmIAA18* were the highest at ED1 and then began to decline (Figure 5B). In addition, the levels of *PmIAA3, PmIAA9,* and *PmIAA14* increased from ED1 to ED2 and then gradually decreased, while the levels of *PmIAA2, PmIAA4,* and *PmIAA18* gradually increased from ED1 to ED3 and then began to decrease (Figure 5B). Compared with the trend of *PmIAA2, PmIAA6, PmIAA7*, *PmIAA11,* and *PmIAA17* decreased firstly from ED1 to ED2 and increased from ED2 to ED3 and then began to decrease (Figure 5B). In general, 14 PmIAAs were relatively high-expressed during the dormancy stage (including ED1, ED2, and ED3). 

### Expression pattern analysis of *PmIAAs* in hormonal and stress responses

**FIGURE 6:**
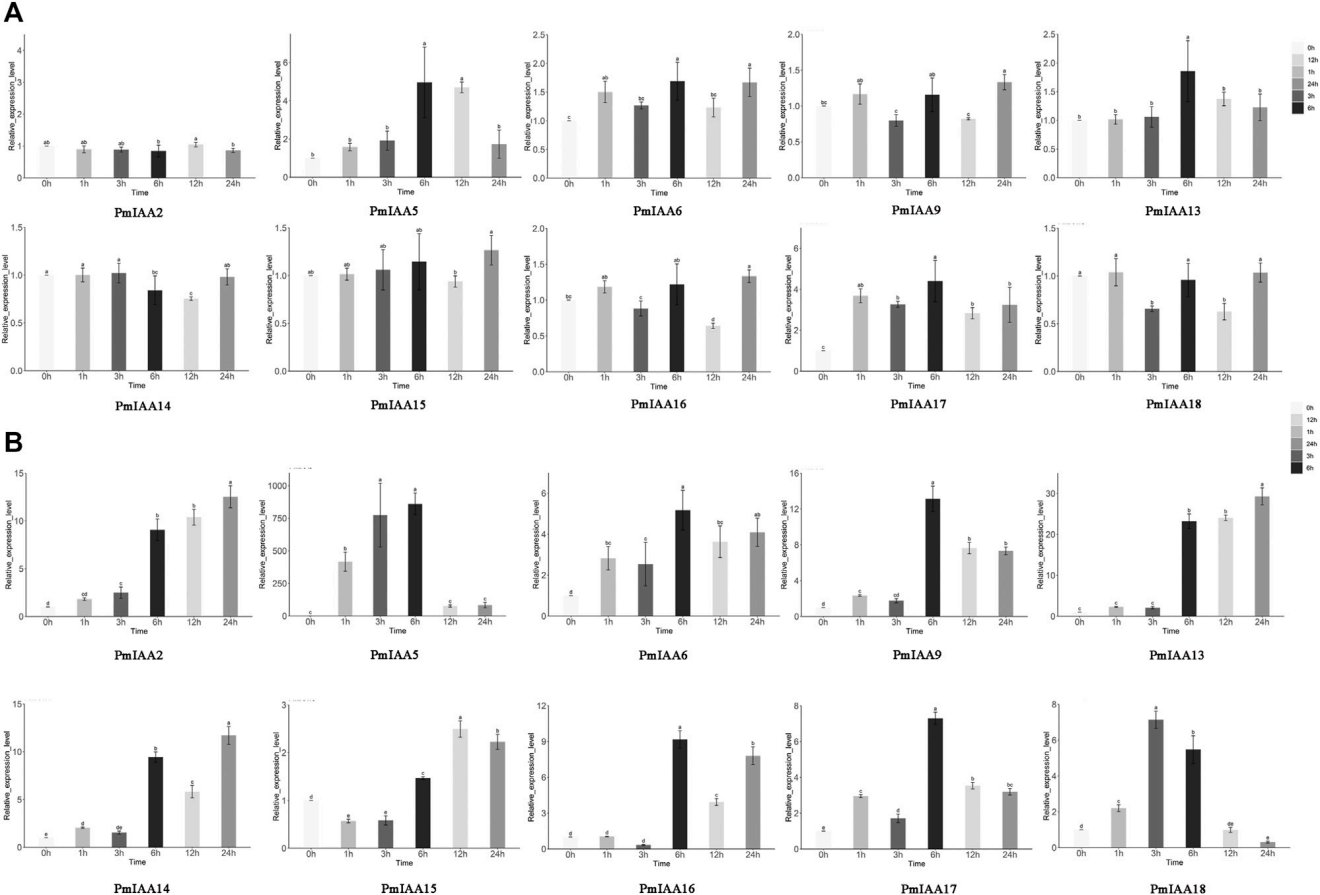
Expression pattern analysis of *PmIAAs* in IAA response **(A)** The relative gene expression level of *PmIAAs* after IAA treatment in buds **(B)** The relative gene expression level of *PmIAAs* after IAA treatment in stems. The mean ± standard error of three replicates is shown. The letters above the error bars are marked with significance for expression levels.

To recognize the functional role of *PmIAA* genes in auxin, drought, and salt stress responses, we selected several *PmIAAs* from each group (a total of six groups) and analyzed their gene expression profiles using qRT-PCR assays (Figure 2). Upon auxin treatment, different *PmIAAs* displayed different expression trends across floral buds, stems and leaves. Among *PmIAAs* from six groups, we observed no significant change in the gene expression level of *PmIAA2, PmIAA9*, and *PmIAA16* from Group Ⅰ and the other four *PmIAAs* (*PmIAA6, PmIAA13, PmIAA14, and PmIAA15*) in floral buds (Figure 6A). In contrast, the expression level of *PmIAA5* from Group Ⅴ and *PmIAA17* from Group Ⅳ reached the maximum value within 6 hours after IAA treatment in floral buds and then began to decline (Figure 6A). In leaves, the expression levels of nine *PmIAAs* (*PmIAA2, PmIAA5, PmIAA6, PmIAA9, PmIAA13, PmIAA14, PmIAA15, PmIAA16,* and *PmIAA17*) changed significantly 1 hour after IAA treatment and reached their maximum level at different time points (Supplementary Figure S3). On the other hand, the expression levels of *PmIAA5*, *PmIAA6*, *PmIAA9*, *PmIAA17*, and *PmIAA18* significantly increased after 1 h of IAA treatment in stems and peaked at 3 h or 6 h (Figure 6B). The expression patterns of *PmIAA2*, *PmIAA13*, and *PmIAA14* maintained a rising trend in stems (Figure 6B). 

**FIGURE 7:**
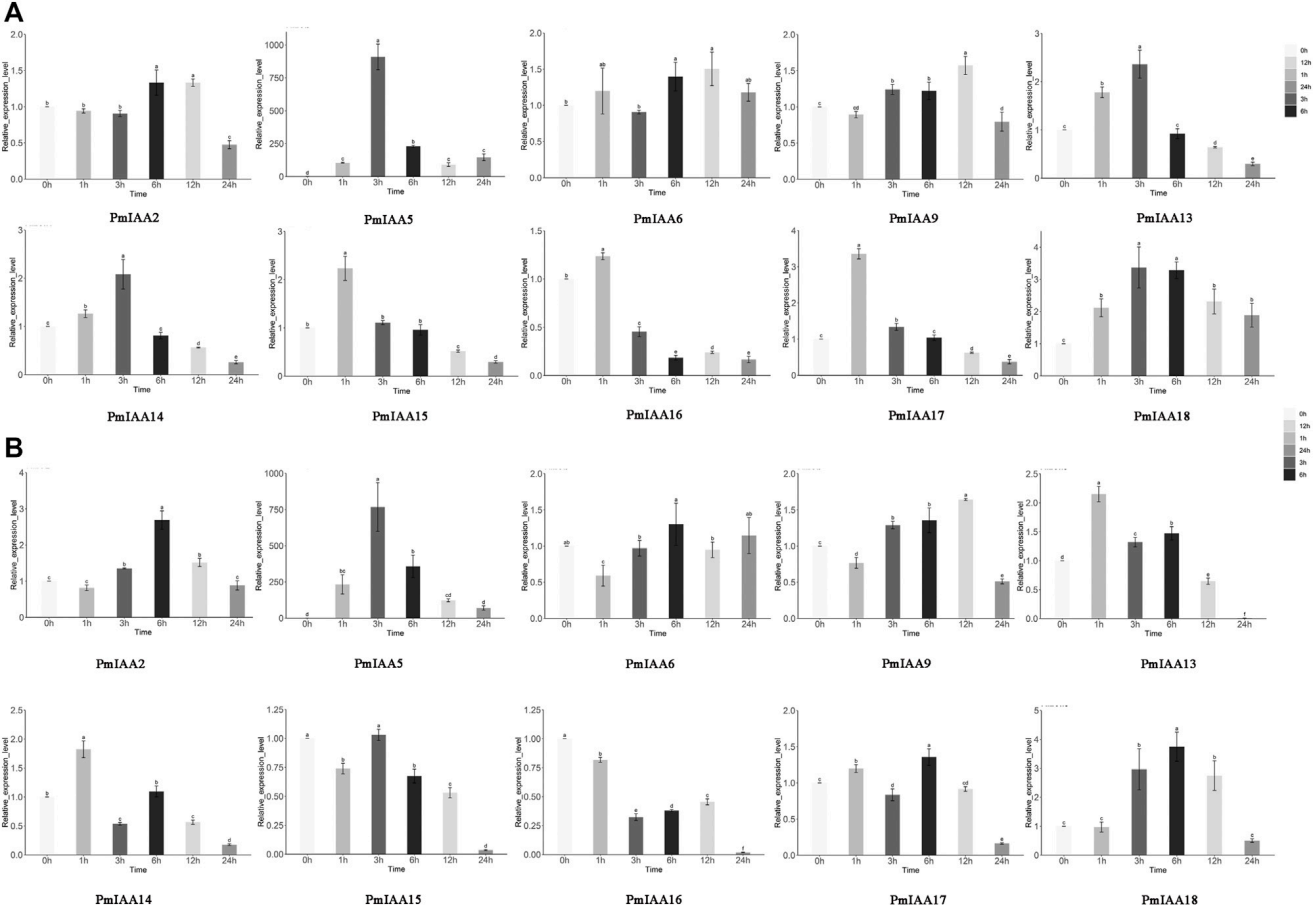
Expression pattern analysis of *PmIAAs* in abiotic stress responses **(A)** The relative gene expression level of *PmIAAs* after drought treatment in stems **(B)** The relative gene expression level of *PmIAAs* after salt treatment in stems. The mean ± standard error of three replicates is shown. The letters above the error bars are marked with significance for expression levels.

Since we detected drought and stress response-related cis-element within *PmIAA* gene promoters, we further examined the expression level patterns of *PmIAA* genes in stem tissues under drought and salt stress treatment. With drought treatment of mei, the expression levels of eight genes (*PmIAA5, PmIAA9, PmIAA13, PmIAA14, PmIAA15, PmIAA16, PmIAA17,* and *PmIAA18*) were increased significantly after 1h to the maximum level and then began to decline (Figure 7A). On the other hand, six genes (*PmIAA2, PmIAA5, PmIAA13, PmIAA14, PmIAA17,* and *PmIAA18*) were highly induced after salt stress treatment (Figure 7B).

### Gene duplication and synteny analysis of Aux/IAA family genes

**FIGURE 8:**
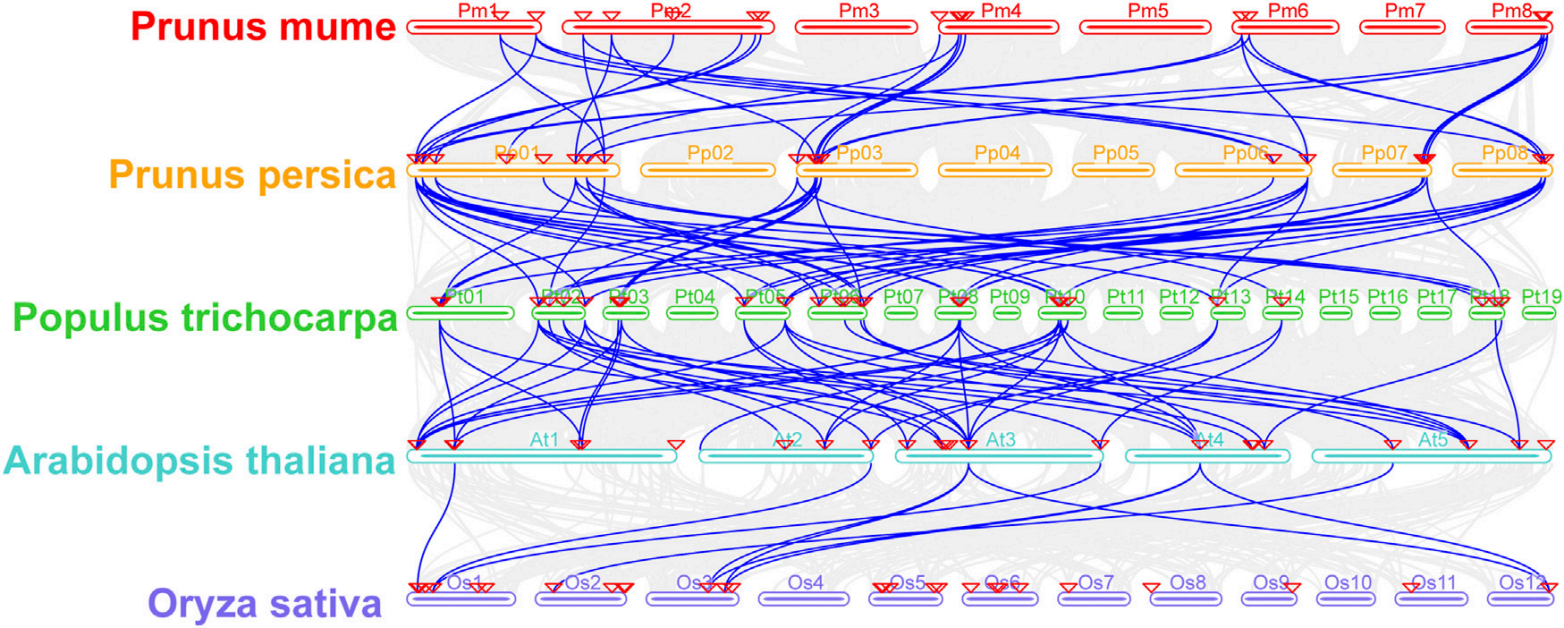
The synteny analysis of IAA genes from mei, Arabidopsis, peach, poplar, and rice. The gray lines in the background represent the collinear blocks among five species, the blue lines emphasize the syntenic *Aux/IAA* gene pairs and the red triangle represents *Aux/IAA* genes.

To study the evolutionary origin of *PmIAA* gene family genes, we first inferred the duplication events of IAA genes from five species, including four dicotyledonous species and one monocotyledonous species, with MCScanX analysis. Nine *PmIAAs* (including *PmIAA6, PmIAA7, PmIAA10, PmIAA12, PmIAA13, PmIAA14, PmIAA17, PmIAA18* and *PmIAA19*) might be arised from WGD or segmental duplication. Eight *PmIAAs* (*PmIAA1, PmIAA2, PmIAA3, PmIAA4, PmIAA5, PmIAA11, PmIAA15* and *PmIAA16*) were classified as dispersed duplication. Only two *PmIAAs* (*PmIAA8* and *PmIAA9*) were possibly originated from tandem duplication (Supplementary Table S19). Furthermore, the genome synteny analysis of poplar, Arabidopsis, peach, mei, and rice generated a total of 166 collinearity gene pairs among five species (Figure 8, Supplementary Table S20). By comparing the 19 *PmIAAs* and 23 *PpIAAs*, eight *PmIAAs* are in one-to-one correspondence with their orthologous genes in peach (Figure 8, Supplementary Table S21). Moreover, we observed six *PpIAAs* having five corresponding *PtIAAs* from poplar (Figure 8, Supplementary Table S22). Furthermore, 27 *PtIAAs* have 24 orthologous genes in Arabidopsis (Figure 8, Supplementary Table S23). However, only seven genes in Arabidopsis have six corresponding genes in rice (Figure 8, Supplementary Table S24).

### Protein interaction network of PmIAA proteins

To understand the genetic interaction relationship among *Aux/IAA* genes in mei, we first identified the Arabidopsis orthologues for *PmIAA* genes by blasting against Arabidopsis. A total of 16 Arabidopsis ortholog proteins corresponding to 19 PmIAA proteins were identified with identity ≥40%. The genetic interaction among 16 AtIAA proteins was analyzed and the regulatory network was constructed based on the STRING database (Supplementary Table 25). We found a total of 108 pairwise interactions from the protein interaction network, among which AtIAA27 is predicted to interact with 15 other AtIAA proteins and AtIAA30 is predicted to interact with eight AtIAAs (Supplementary Figure S26).

## Discussion

Auxin is extremely vital for regulating the life activities of plants. ARFs and Aux/IAAs are involved in auxin signaling transduction. Earlier reports have proposed that *Aux/IAA* family genes are necessary to regulate diverse plant developmental processes. The molecular mechanism of *Aux/IAA* has been thoroughly elaborated in plant model systems, especially in Arabidopsis and rice (Guilfoyle, 2015). In woody perennials, researchers were mainly focused on studying the effect of *Aux/IAA* genes in regulating secondary growth in trees (Groover, 2005). During cambium development, DELLA protein RGL1 (REPRESSOR of ga1-3 Like 1), ARF7, and IAA9 can form terpolymer complexes that facilitate the crosstalk between auxin and gibberellin signal pathways (Hu et al., 2022). Then again, the Aux/IAA-ARF signal cascade is also involved in the secondary xylem development of poplar by affecting HD-ZIP III transcription factors (Xu et al., 2019). In poplar, the bZIP53-IAA4 complex was found to negatively regulate the adventitious root development and can respond to salt stress (Zhang et al., 2020). Nevertheless, few studies focused on the role of *Aux/IAA* genes in controlling reproductive growth in woody plants.

To understand the function of *Aux/IAA* genes in mei, we characterized 19 *PmIAAs* and systematically investigated their gene structure, protein sequence conservation, evolutionary path, and regulatory model. In total, we identified 19 *PmIAAs* in mei, which is similar to 23 *PpIAAs* detected in peach (Guan et al., 2019). However, the number of *Aux/IAA* genes was significantly reduced compared with 29 in Arabidopsis and 35 in poplar. This is likely due to fewer gene duplication events occurring in the mei genome compared with that in other plant species. For example, we observed that 28 *PtIAAs* arose from segmental duplication or the recent salicoid WGD event and 15 *PtIAAs* were classified as tandem duplication (Kalluri et al., 2007). While only eight *PmIAAs* originated from WGD or segmental duplication. Furthermore, we observed that the number and grouping of *Aux/IAA* genes between Arabidopsis and rice and other woody plants are relatively large. And *PmIAAs* have the highest homology to *PpIAAs* and the lowest homology to *OsIAAs,* which is consistent with the species phylogeny among the investigated plant species.

Based on the protein sequence alignment between PmIAA proteins and PpIAA proteins, PmIAA proteins were relatively conserved, especially in the domain Ⅲ and domain Ⅳ. However, five PmIAAs and three PmIAAs lacked domain I and domain II, respectively. For example, PmIAA6, the mei ortholog of AtIAA30 in Arabidopsis, lacked both domains I and domain II, indicating that PmIAA6 is likely one of the non-canonical Aux/IAA proteins. This result is consistent with previous reports that AtIAA30 lacked the domain II in *Arabidopsis* (Dreher et al., 2006; Wu et al., 2012). Preceding reports have revealed that domain I contains the ‘LxLxLx’ motif, which can recruit TPL proteins to repress the transcriptional activity of downstream *ARF* genes (Szemenyei et al., 2008). In addition, domain II was important for the degradation of Aux/IAA protein. Therefore, these PmIAA proteins lacking domain II, may escape the recognition of TIR1/AFB receptors due to deletion of domain II, and might be regulated by TMK1 or MPK14 in auxin signaling pathways.

The PmIAA proteins were classified into six groups and the classification was related to their motif composition and primary sequences. Although PmIAA proteins have a highly conserved Aux/IAA domain, variations in the structure of PmIAA proteins may lead to functional differentiation. For example, though *PmIAA9* and *PmIAA16* share 64.92% protein identity, their expression profiles were highly varied. The expression levels of *PmIAA9* were high in floral buds at the endodormancy stage, while *PmIAA16* was highly expressed in stems and flushed floral buds (Figures 5A,B). Among 19 *PmIAAs*, nine *PmIAAs* were highly expressed in the buds and the stems respectively, indicating their possible role in the bud and stem development. Furthermore, 14 *PmIAAs* had relatively high expression levels at the dormancy release process, suggesting that they may play a role in regulating dormancy cycling and flower bud development. Additionally, we observed auxin responsive elements (TGA-element and AuxRR-core element), gibberellin responsive elements (P-box, TATC-box, and GARE-motif), and salicylic acid (TCA-element) responsive elements within the promoters of *PmIAA* genes, indicating their possible involvement in hormonal responses. With exogenous auxin treatment, the overall gene expression level of four *PmIAAs* (*PmIAA5, PmIAA6*, *PmIAA9,* and *PmIAA18*) in the stem were increased reaching the maximum value within three to 6 hours, then began to decline. The expression trend of *PmIAAs* was consistent with that of *Aux/IAA* genes in soybean after auxin treatment (Hagen and Guilfoyle, 1985). *PmIAA5, PmIAA6*, *PmIAA9,* and *PmIAA18* are likely involved in stem development. In general, the differentiated expression pattern of *PmIAA* genes across different tissues suggested their divergent functional role in regulating flower bud, fruit, stem, and root development in mei.

The expression pattern analysis of *PmIAAs* after stress treatment indicated that *PmIAAs* might also be engaged in the abiotic stress-responsive processes. Firstly, we detected numerous stress-responsive *cis*-acting elements on *PmIAA* gene promoters (Figure 4), such as the ABRE element involved in abscisic acid-regulated osmotic stress (Kim et al., 2011), TGACG-motif and CGTCA-motif involved in the secondary metabolism and stress responses (Wang et al., 2019). In addition, we also detected drought response, low-temperature responsive, and wounding response elements within *PmIAA* gene promoters. The expression pattern analysis also confirmed the induced transcription of *PmIAAs* upon drought and salt treatment. Previous studies reported that the expression of *Aux/IAA* genes were induced to increase after drought and salt treatments (He et al., 2018). Furthermore, we observed that the relative expression levels of seven *PmIAA* genes changed significantly after treatments. These seven *PmIAAs* (including *PmIAA5, PmIAA13, PmIAA14, PmIAA15, PmIAA16, PmIAA17,* and *PmIAA18*) might respond to drought stress. We observed eight *PmIAA* genes that may respond to salt stress. The expression levels of five *PmIAAs* (*PmIAA2*, *PmIAA5*, *PmIAA13*, *PmIAA14*, and *PmIAA18*) firstly increased and then decreased, which is parallel to the expression of their *Aux/IAA* orthologous in apple (Li et al., 2022). While the expression levels of *PmIAA15 and PmIAA16* showed a gradually decreasing trend after treatments with salt, which is different from the expression patterns of their orthologs reported by former researchers (Li et al., 2022). The differentiated expression pattern may result from their functional divergence during evolution. Interestingly, we detected four genes (*PmIAA5, PmIAA13, PmIAA14, PmIAA18*) responsive to both drought stress and salt stress, indicating their role in regulating both drought and salt stress responses in mei. Future studies can further clarify the functional mechanisms of the *PmIAA* genes during floral bud development, abiotic stress responses, and hormonal signaling in mei with molecular experiments and other related technologies.

## Conclusion

In this study, we identified 19 PmIAA proteins across the genome of *P. mume* and analyzed gene structure, protein features, and promoter sequences, and explored their potential roles in regulating floral bud development and stress response in mei*.* With bioinformatics analysis, we observed that the 19 PmIAA proteins contained four classical domains and can be divided into six groups based on phylogenetic analysis. With tissue-specific expression analysis, *PmIAAs* were relatively high-expressed in floral buds and stems. Moreover, we identified 14 PmIAAs with relatively high expression levels during the dormancy release process, implicating their possible role in regulating dormancy cycling and flower development. Furthermore, based on the differential expression pattern of *PmIAAs* after auxin, drought, and salt stress treatment, *PmIAA5, PmIAA13, PmIAA14,* and *PmIAA18* may be implicated in regulating drought and salt stress response. *PmIAA5* and *PmIAA17* are possibly involved in regulating the auxin response in buds, leaves, and stems*.* In general, *PmIAAs* are essential to floral bud and stem development, auxin response, and abiotic stress responses in *P. mume*. Our study provides insights into the evolution and functional roles of *PmIAA* genes in *P. mume*, which will facilitate the understanding of *Aux/IAA* family genes in relevant biological processes in perennial woody plants.

## Data Availability

The datasets for this study can be found in the article or Supplementary Material. Further requirements can be directed to the corresponding authors.
