# Corrigendum: Genome-wide identification of Aux/IAA gene family and their expression analysis in *Prunus mume*

**DOI:** 10.3389/fgene.2023.1208488

**Published:** 2023-05-09

**Authors:** Wenhui Cheng, Man Zhang, Tangren Cheng, Jia Wang, Qixiang Zhang

**Affiliations:** ^1^ Beijing Key Laboratory of Ornamental Plants Germplasm Innovation and Molecular Breeding, National Engineering Research Center for Floriculture, School of Landscape Architecture, Beijing Forestry University, Beijing, China; ^2^ Beijing Laboratory of Urban and Rural Ecological Environment, Engineering Research Center of Landscape Environment of Ministry of Education, School of Landscape Architecture, Beijing Forestry University, Beijing, China; ^3^ Key Laboratory of Genetics and Breeding in Forest Trees and Ornamental Plants of Ministry of Education, School of Landscape Architecture, Beijing Forestry University, Beijing, China

**Keywords:** Aux/IAA gene family, *Prunus mume*, evolutionary analysis, expression pattern analysis, auxin-responsive genes

In the published article, there was an error in **Affiliation**. The affiliation for the authors was written incorrectly. The wrong affiliation read as: “Key Laboratory of Genetics and Breeding in Forest Trees and Ornamental Plants of Ministry of Education, School of Landscape Architecture, Beijing Forestry University, Beijing, China”. The correct affiliation should read as: “Beijing Key Laboratory of Ornamental Plants Germplasm Innovation & Molecular Breeding, National Engineering Research Center for Floriculture, Beijing Laboratory of Urban and Rural Ecological Environment, Engineering Research Center of Landscape Environment of Ministry of Education, Key Laboratory of Genetics and Breeding in Forest Trees and Ornamental Plants of Ministry of Education, School of Landscape Architecture, Beijing Forestry University, Beijing, China”.

The authors apologize for this error and state that this does not change the scientific conclusions of the article in any way. The original article has been updated.

