# Supplementary material for: Genome-wide identification of Aux/IAA gene family and their expression analysis in Prunus mume
Source: Front Genet. 2022 Oct 12;13:1013822. doi: 10.3389/fgene.2022.1013822 (PMC9597081; doi:10.3389/fgene.2022.1013822)
Supplement: Supplementary file 1 [file DataSheet1.ZIP › Supplementary/Supplementary_Material.docx]

Supplementary Material

# Supplementary Figures and Tables

## Supplementary Figures

**Supplementary Figure 1.** Chromosome localization of PmIAA genes. PmIAA genes were located across five chromosomes. Pm1, 2, 4, 6, 8 represent chromosomes 1, 2, 4, 6, and 8 of mei, respectively. Chromosomes three, five, and seven have no PmIAA genes. The red line indicates the duplicated gene pair.

**Supplementary Figure 2.** Different protein motifs sequence from Motif 1 to Motif 9 identified using MEME.

**Supplementary Figure 3.** The relative gene expression level of *PmIAAs* after IAA treatment in leaves. The mean ± standard error of three replicates is shown. The letters above the error bars are marked with significance for expression levels.

**Supplementary Figure 4.** Protein interaction networks of PmIAA proteins. The nodes with different colors represent different genes and the lines with different colors represent different types of interaction.

**Supplementary Figure 5.** The relative gene expression level of PmIAAs during diverse tissues and dormancy periods. The mean ± standard error of three replicates is shown. The letters above the error bars are marked with significance for expression levels.

## Supplementary Tables

**Supplementary Table 1.** Primers used for qRT-PCR analysis.

**Supplementary Table 2.** Whole-genome classification of 19 *PmIAA* genes and their protein features.

**Supplementary Table 3.** Identity of pairwise alignment within PmIAA proteins.

**Supplementary Table 4.** Protein sequences of Arabidopsis, poplar, mei, peach and rice.

**Supplementary Table 5.** The information and predicated fuctions of motif sequence

**Supplementary Table 6.** Count of cis-acting elements in the PmIAA genes.

**Supplementary Table 7.** The specific location and sequence of cis-acting elements in the PmIAA genes.

**Supplementary Table 8.** List of PmIAA genes along-with their gene duplication types.

**Supplementary Table 9.** The collinearity genelink of Aux/IAA family genes in mei, Arabidopsis, peach, rice and poplar genomes.

**Supplementary Table 10.** *PmIAAs* of *Prunus mume* and their corresponding homologous genes in *Arabidopsis thaliana*
