# Supplementary figures and images for: Genome-wide identification of Aux/IAA gene family and their expression analysis in Prunus mume
Source: Front Genet. 2022 Oct 12;13:1013822. doi: 10.3389/fgene.2022.1013822 (PMC9597081; doi:10.3389/fgene.2022.1013822)

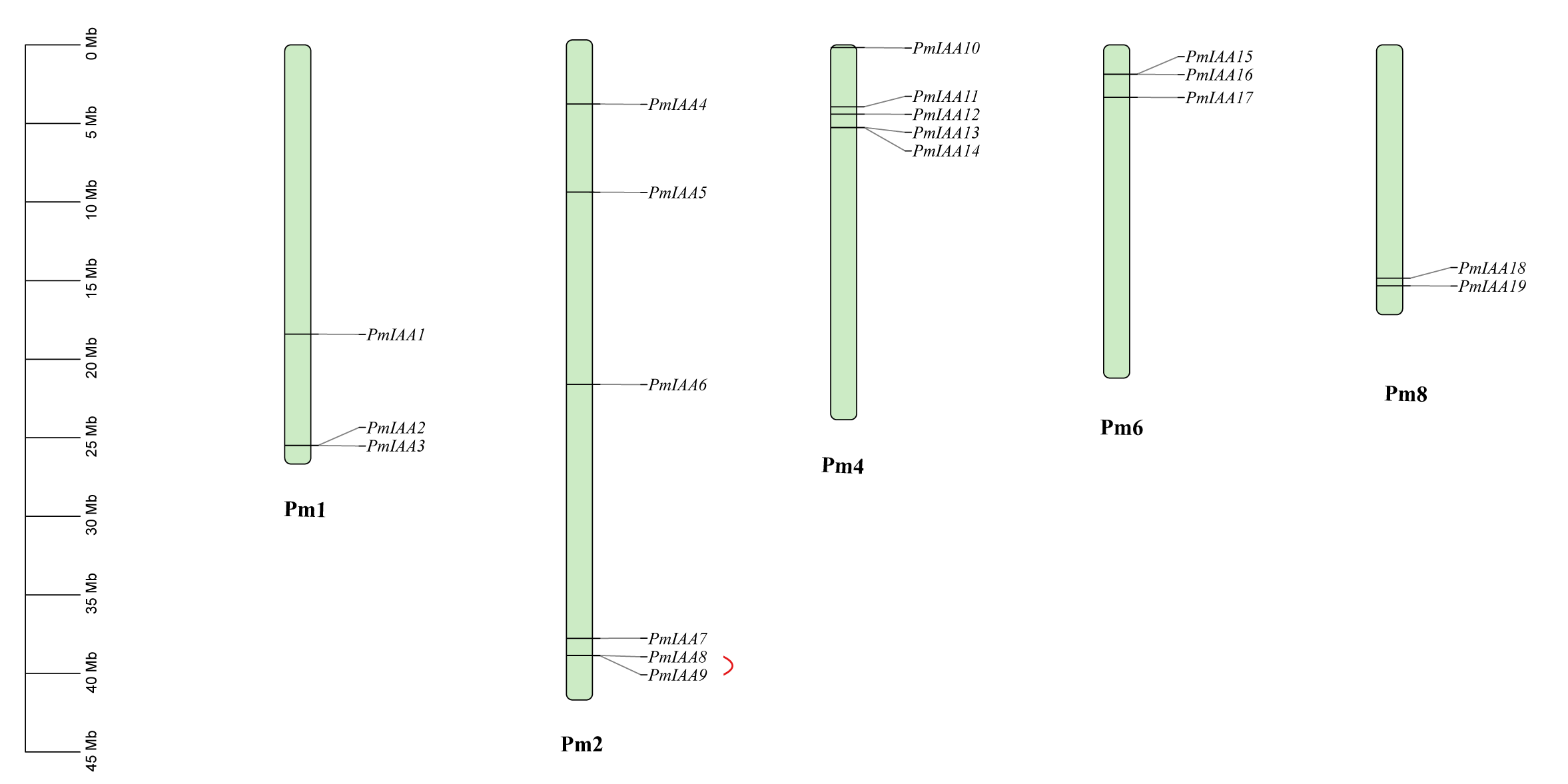

Supplement: Supplementary file 1 [file DataSheet1.ZIP › Supplementary/Supplementary Figure 1.tif]

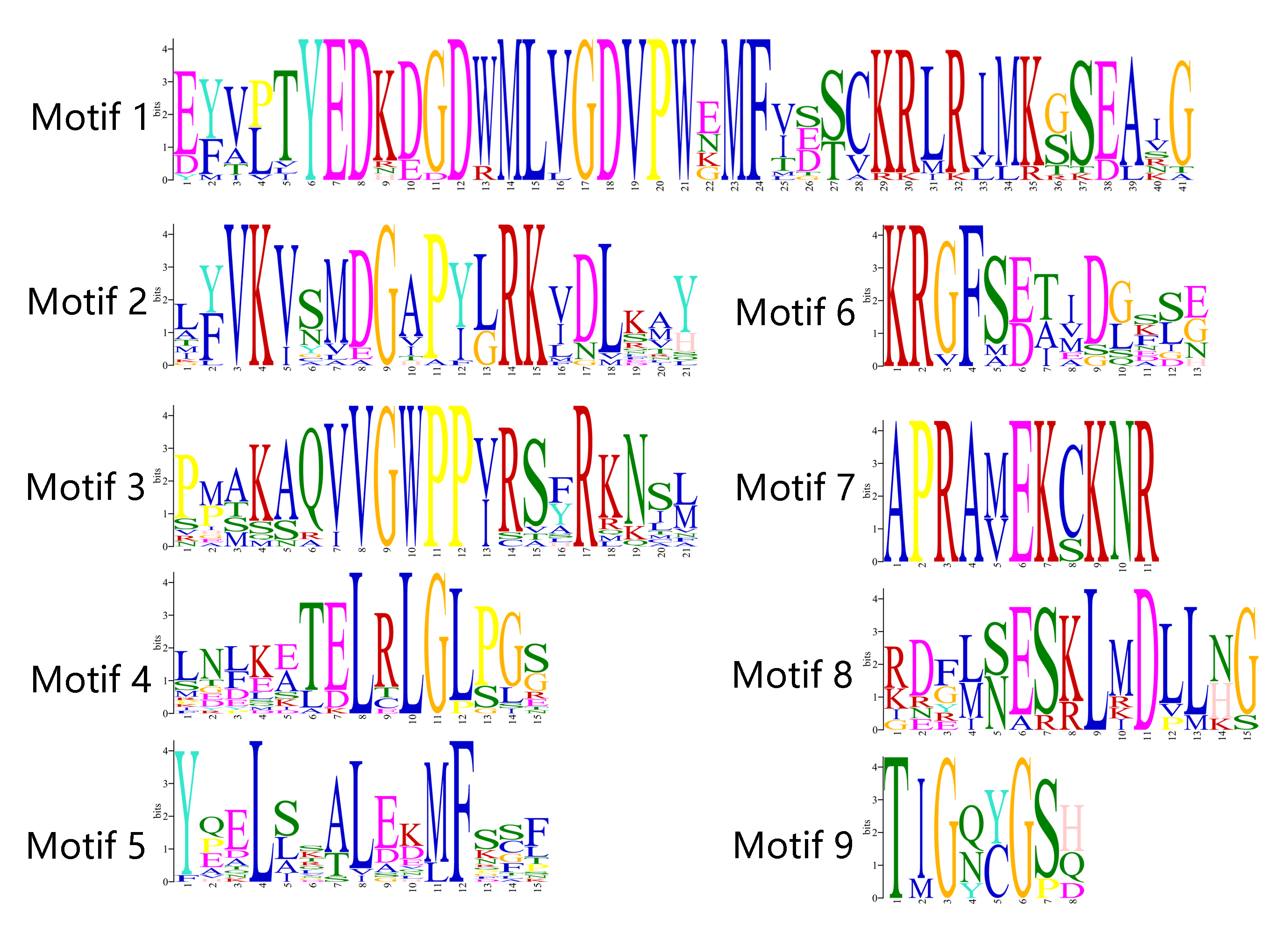

Supplement: Supplementary file 1 [file DataSheet1.ZIP › Supplementary/Supplementary Figure 2.tif]

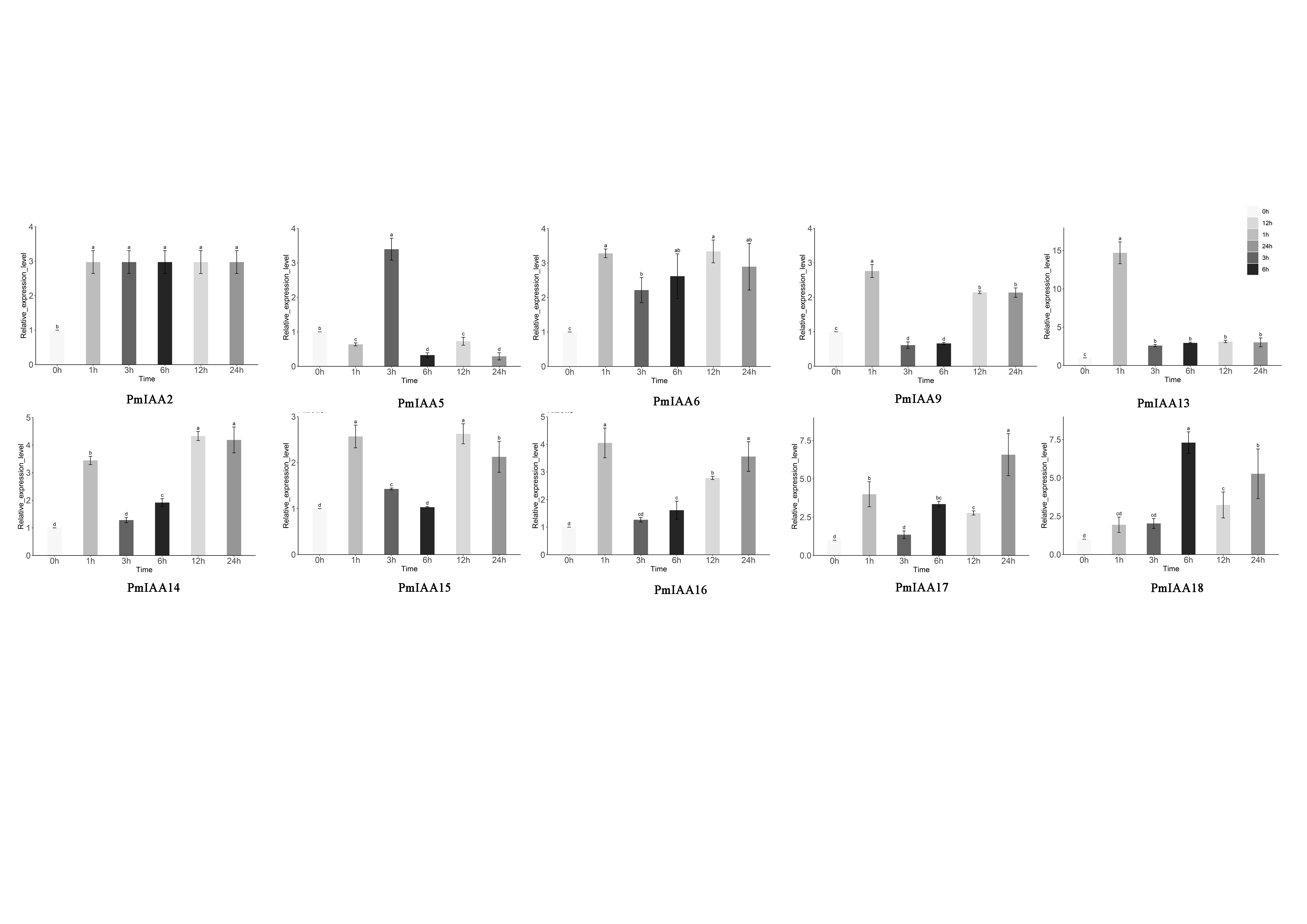

Supplement: Supplementary file 1 [file DataSheet1.ZIP › Supplementary/Supplementary Figure 3.tif]

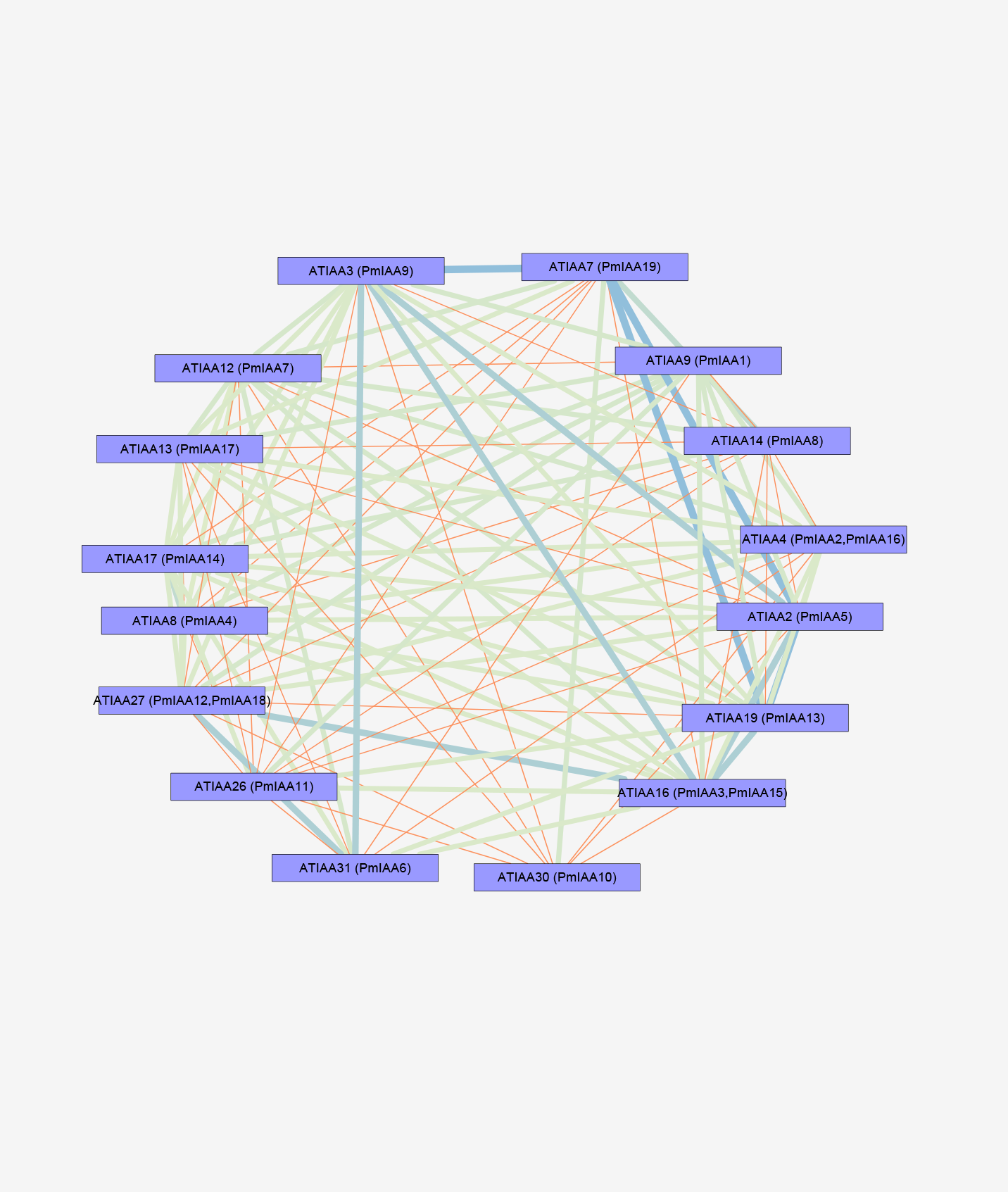

Supplement: Supplementary file 1 [file DataSheet1.ZIP › Supplementary/Supplementary Figure 4.tif]

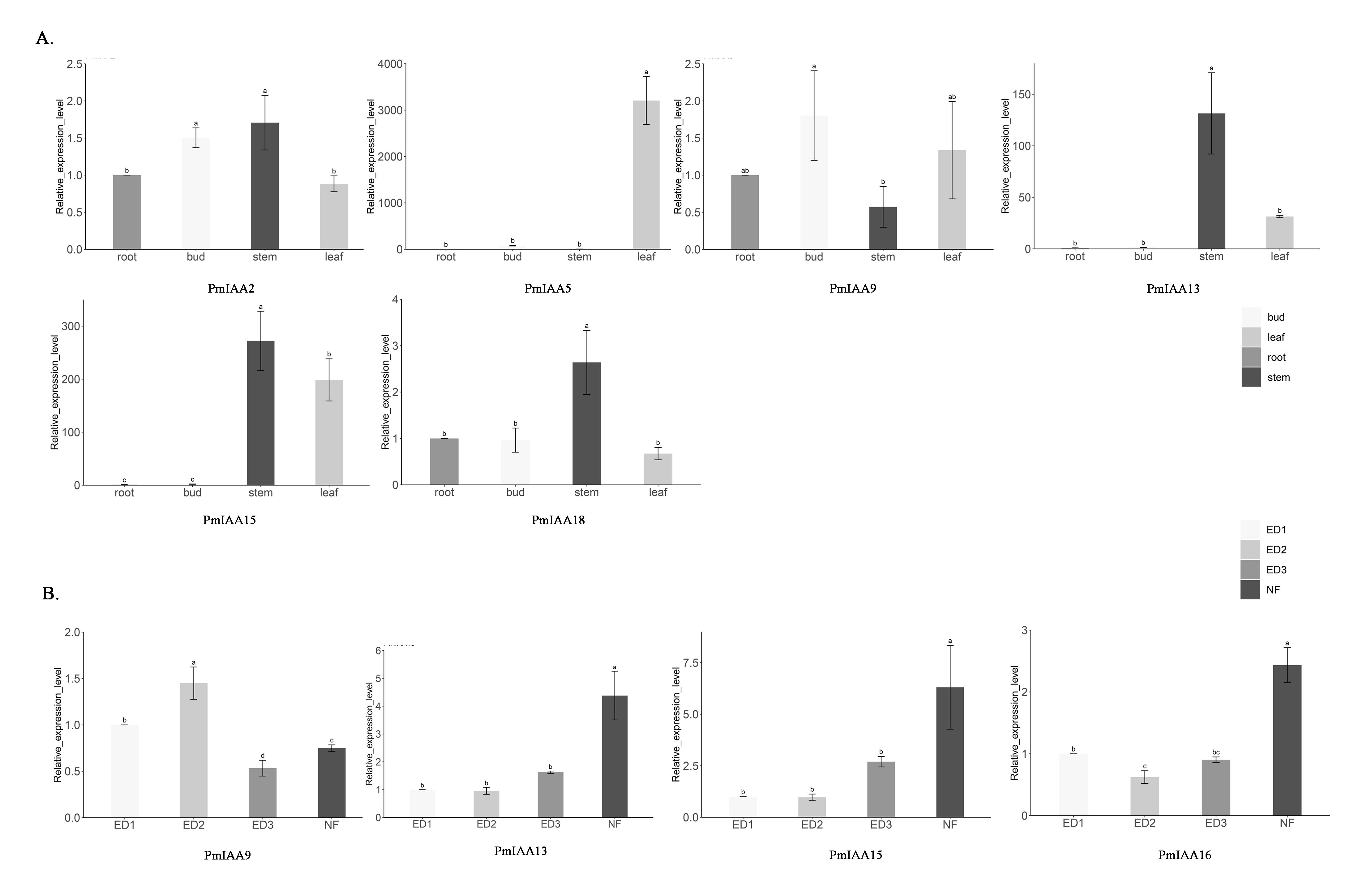

Supplement: Supplementary file 1 [file DataSheet1.ZIP › Supplementary/Supplementary Figure 5.tif]
